# Tmem161a regulates bone formation and bone strength through the P38 MAPK pathway

**DOI:** 10.1038/s41598-023-41837-4

**Published:** 2023-09-05

**Authors:** Takuya Nagai, Tomohisa Sekimoto, Syuji Kurogi, Tomomi Ohta, Shihoko Miyazaki, Yoichiro Yamaguchi, Takuya Tajima, Etsuo Chosa, Mai Imasaka, Kumiko Yoshinobu, Kimi Araki, Masatake Araki, Narantsog Choijookhuu, Katsuaki Sato, Yoshitaka Hishikawa, Taro Funamoto

**Affiliations:** 1 Division of Orthopaedic Surgery, Department of Medicine of Sensory and Motor Organs, Faculty of Medicine, University of Miyazaki, 5200 Kihara, Kiyotake, Miyazaki 889-1692 Japan; 2 Department of Genetics, Hyogo Medical University, Nishinomiya, Japan; 3 Institute of Resource Development and Analysis, Kumamoto University, Kumamoto, Japan; 4 Department of Anatomy, Histochemistry and Cell Biology, Faculty of Medicine, University of Miyazaki, Miyazaki, Japan; 5 Division of Immunology Department of Infectious Disease, Faculty of Medicine, University of Miyazaki, Miyazaki, Japan

**Keywords:** Bone, Osteoblasts

## Abstract

Bone remodeling is an extraordinarily complex process involving a variety of factors, such as genetic, metabolic, and environmental components. Although genetic factors play a particularly important role, many have not been identified. In this study, we investigated the role of transmembrane 161a (Tmem161a) in bone structure and function using wild-type (WT) and Tmem161a-depleted (Tmem161a^GT/GT^) mice. Mice femurs were examined by histological, morphological, and bone strength analyses. Osteoblast differentiation and mineral deposition were examined in Tmem161a-overexpressed, -knockdown and -knockout MC3T3-e1 cells. In WT mice, Tmem161a was expressed in osteoblasts of femurs; however, it was depleted in Tmem161a^GT/GT^ mice. Cortical bone mineral density, thickness, and bone strength were significantly increased in Tmem161a^GT/GT^ mice femurs. In MC3T3-e1 cells, decreased expression of alkaline phosphatase (ALP) and Osterix were found in Tmem161a overexpression, and these findings were reversed in Tmem161a-knockdown or -knockout cells. Microarray and western blot analyses revealed upregulation of the P38 MAPK pathway in Tmem161a-knockout cells, which referred as stress-activated protein kinases. ALP and flow cytometry analyses revealed that Tmem161a-knockout cells were resistant to oxidative stress. In summary, Tmem161a is an important regulator of P38 MAPK signaling, and depletion of Tmem161a induces thicker and stronger bones in mice.

## Introduction

Bone homeostasis is maintained by bone remodeling, which involves bone resorption by osteoclasts and subsequent bone formation by osteoblasts. Osteoblasts are regulated by a variety of genes and transcription factors^1^. For example, the P38 MAPK-osterix (OSX) pathway plays an important role in the regulation of osteogenesis-related gene expression^2,3^. Moreover, excessive oxidative stress reduces osteoblast differentiation, survival, and bone formation, which in turn can result in osteoporosis^4,5^. Osteoporosis can lead to fragility fractures, such as proximal femur fractures and vertebral fractures, resulting in significant morbidity and mortality^6,7^. In recent years, osteoporosis treatments have been targeted to inhibit bone resorption using bisphosphonates and promote bone formation using teriparatide and anti-sclerostin antibodies^8^. Although new treatments are being introduced into clinical practice, multiple genetic factors and the complex pathogenesis are still become challenges in the effective treatment for osteoporosis. Therefore, it is essential to understand the genetic factors involved in bone remodeling.

We recently designed a new screening system to identify and analyze the function of novel genes involved in bone metabolism using mutant mouse strains registered in the Exchangeable Gene Trap Clones database^9,10^. The principle of gene trapping involves randomly inserting a trap vector containing a drug-resistance gene into the embryonic stem cell genome and disrupting only target genes with the inserted vector. The advantage of this method compared with other methods is enhanced efficiency for novel gene-mutant mouse production^9^. In this screening, Tmem161a was selected as a candidate gene for analysis because there were no previous reports on bone metabolism and it had high bone and joint scores in EST profile.

Tmem161a, also known as AROS-29 (adaptive response to oxidative stress), was first reported in 2006^11^. Expression of Tmem161a in Saos-2 cells was shown to be associated with strong resistance to oxidant-induced DNA damage and apoptosis. In addition, Tmem161a is reportedly a tumor-associated antigen overexpressed in human non-small cell lung cancer, and it is related to specific gene expression signatures such as *MYC* and cell cycle genes^12^. Although it has been studied in cancer cells, the physiological role of Tmem161a in bone remodeling remains poorly understood.

In the present study, we first investigated the functional role of Tmem161a in mouse osteoblast differentiation. Bone morphometric and strength analyses were performed in wild-type (WT) and Tmem161a-deficient (*Tmem161a*^*GT/GT*^) mice. Immunohistochemistry studies demonstrated Tmem161a expression in osteoblasts of cortical bones. The functional importance of Tmem161a in osteoblast differentiation was confirmed using MC3T3-e1 murine osteoblasts. Markers of osteoblast differentiation and mineral deposition were examined under various conditions, such as Tmem161a overexpression, knockdown, and knockout (KO). Finally, microarray analyses and oxidative stress induction experiments were performed to elucidate the role of Tmem161a in the molecular mechanism of osteoblast differentiation.

## Results

### Expression of Tmem161a in mouse bone

**Figure 1:**
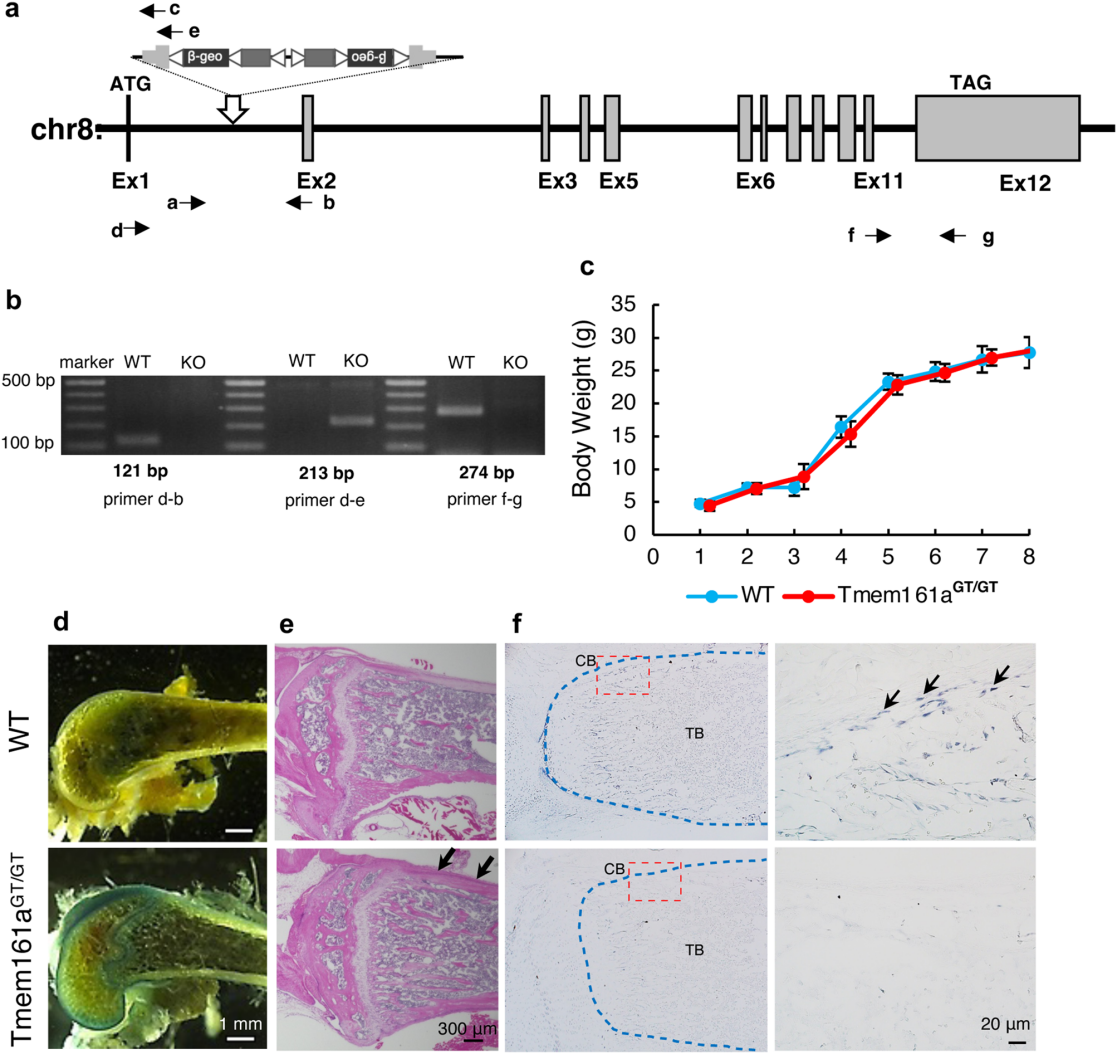
Generation of *Tmem161a*^*GT/GT*^ mice. (**a**) Map of the *Tmem161a*^*GT*^ allele. Trap vectors; two pU21-T with the latter inversion were inserted in intron 1 of *Tmem161a*. (**b**) RT-PCR analysis of *Tmem161a* in WT and *Tmem161a*^*GT/GT*^ mouse bone tissues. Left band is between Exon 1 and Exon 2 (primer d and b). Central band is between Exon 1 and the trap vector (primer d and e). Right band is between Exon 11 and Exon 12 (primer f and g). (**c**) Growth curves of WT and *Tmem161a*^*GT/GT*^ mice. Data represent the mean ± SD from 5 male mice. (**d**) X-gal staining of 8-week-old WT and *Tmem161a*^*GT/GT*^ mouse femurs. Scale bar 1 mm. (**e**) HE staining of 8-week-old WT and *Tmem161a*^*GT/GT*^ mouse femurs. Scale bar 300 μm. (**f**) Immunohistochemistry analysis using with Tmem161a Antibody of 8-day-old mouse femurs. Scale bar 20 μm. *CB* cortical bone, *TB* trabecular bone.

*Tmem161a*^*GT/GT*^ mice were generated by insertion of two pU-21T exchangeable gene trap vectors. A schematic diagram illustrating the insertion points of the gene trap vectors is shown in Fig. 1a. Genomic PCR is shown in the supplementary Fig. S1a. In *Tmem161a*^*GT/GT*^ mice, the vectors were inserted in homozygously, and RT-PCR confirmed that Tmem161a was null. Expression between Exon 1 and Exon 2 sequence (primer d and b) was detected in WT but not *Tmem161a*^*GT/GT*^ mice (Fig. 1b, supplementary Fig. S1b). In contrast, the pU-21T-targeted sequence (primer d and e) was detected in *Tmem161a*^*GT/GT*^ but not WT mice. *Tmem161a*^*GT/GT*^ mice grew normally, with body weight and other phenotypes indistinguishable from their WT littermates (Fig. 1c). Birth rate was consistent with Mendelian ratio and there were no embryonic lethality of *Tmem161a*^*GT/GT*^ mice. The gene encoding beta-galactosidase was inserted into the pU-21T gene trap vector, enabling tissue-specific detection of *Tmem161a* expression by X-gal staining*.* We first screened for *Tmem161a* expression in various tissues (Supplementary Fig. S2). Tissues of WT mice did not exhibit staining, except for gastrointestinal tract tissues, which have high endogenous β-galactosidase activity^13^. In *Tmem161a*^*GT/GT*^ mice, X-gal staining revealed expression of *Tmem161a* in various tissues, including brain, kidney, and bone. Detailed analyses of the bones of *Tmem161a*^*GT/GT*^ mice revealed strong expression in articular cartilage and cortical bones (Fig. 1d). Hematoxylin–eosin (HE) staining revealed thickening of cortical bone in *Tmem161a*^*GT/GT*^ mice comparing with WT mice (Fig. 1e, arrows). Immunohistochemistry analyses showed that Tmem161a was expressed in pericortical osteoblasts as well as the growth plate in femoral bone of WT mice (Fig. 1f). Complete depletion of Tmem161a was confirmed in *Tmem161a*^*GT/GT*^ mice.

### Increased bone strength in *Tmem161a*^*GT/GT*^ mice

**Figure 2:**
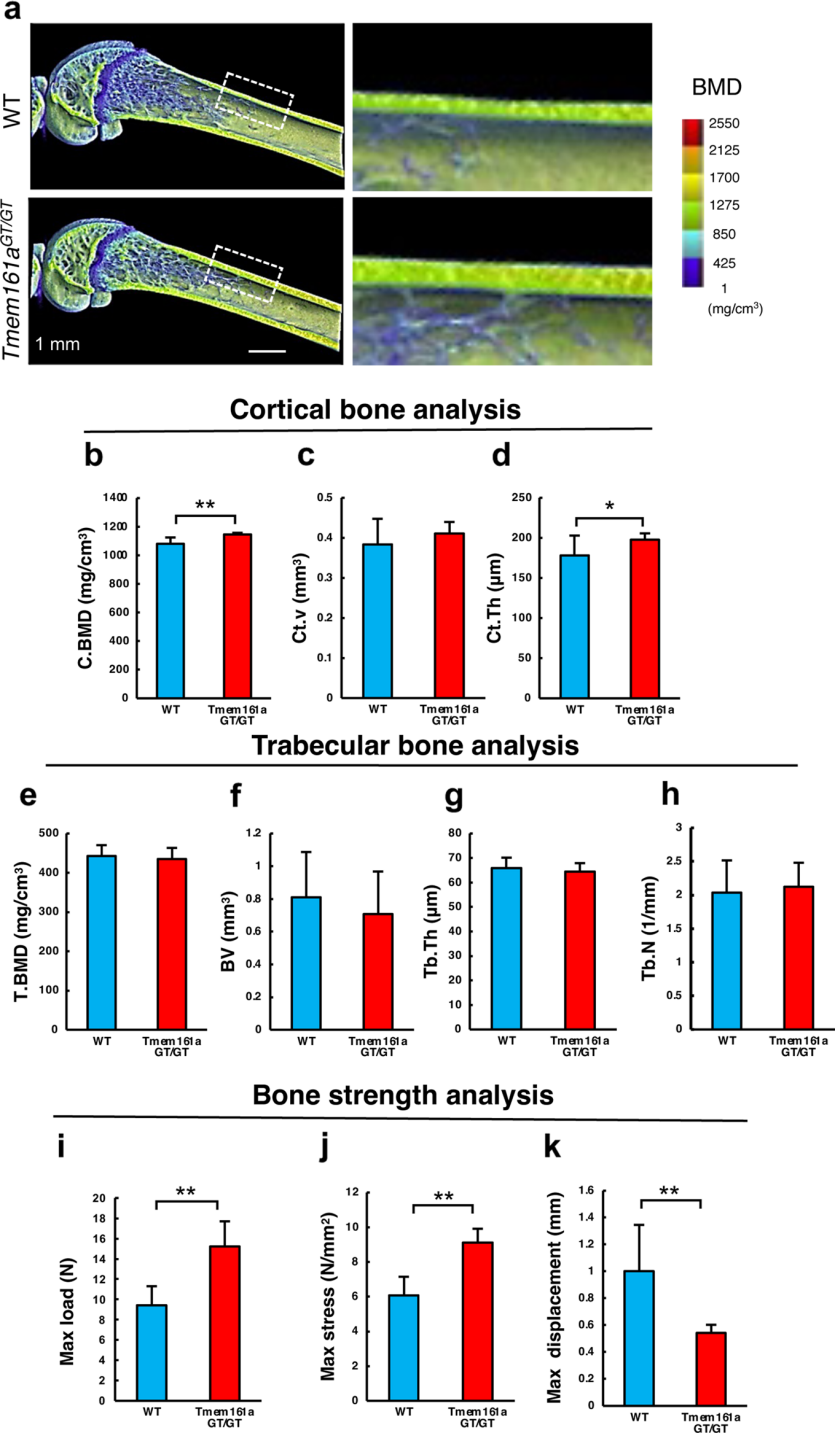
Bone morphometric analysis. (**a**) BMD image of 8-week-old WT and *Tmem161a*^*GT/GT*^ mice femurs. Scale bar 1 mm. Cortical bone mineral density: C.BMD (**b**), Cortical bone volume: Ct.V, (**c**), Cortical bone thickness: Ct.th (**d**), Trabecular bone mineral density: T.BMD (**e**), Trabecular bone volume: BV (**f**). Trabecular bone thickness: Tb.Th (**g**), Trabecular bone number: Tb.N (**h**). Data represent the mean ± SD from 10 mice in each genotype. Biomechanical strength analysis: Max load (**i**), Max stress (**j**), Max displacement (**k**). Data represent the mean ± SD from 5 mice in each genotype. Asterisks indicate statistically significant differences (*p < 0.05, **p < 0.01).

To better understand bone morphology, micro-computed tomography (μCT) scanning was performed on femurs of 8-week-old WT and *Tmem161a*^*GT/GT*^ male mice. In the femurs of *Tmem161a*^*GT/GT*^ mice, thickened cortical bone and higher bone mineral density (BMD) were observed compared with WT littermates (Fig. 2a). Cortical bone parameters, such as cortical BMD (C.BMD) and cortical bone thickness (Ct.Th) were significantly increased in *Tmem161a*^*GT/GT*^ mice (Fig. 2b–d). In contrast, no changes were observed in trabecular bone parameters (Fig. 2e–h). Furthermore, bone strength analyses showed a significant increase in max load and stress in *Tmem161a*^*GT/GT*^ mice (Fig. 2i,j). Moreover, maximum displacement was significantly decreased in the femurs of *Tmem161a*^*GT/GT*^ mice (Fig. 2k). Collectively, these results indicate that *Tmem161a*^*GT/GT*^ mice had significantly stronger bone than WT mice due to thickening of the cortical bone.

### Overexpression of Tmem161a reduces osteoblast differentiation

**Figure 3:**
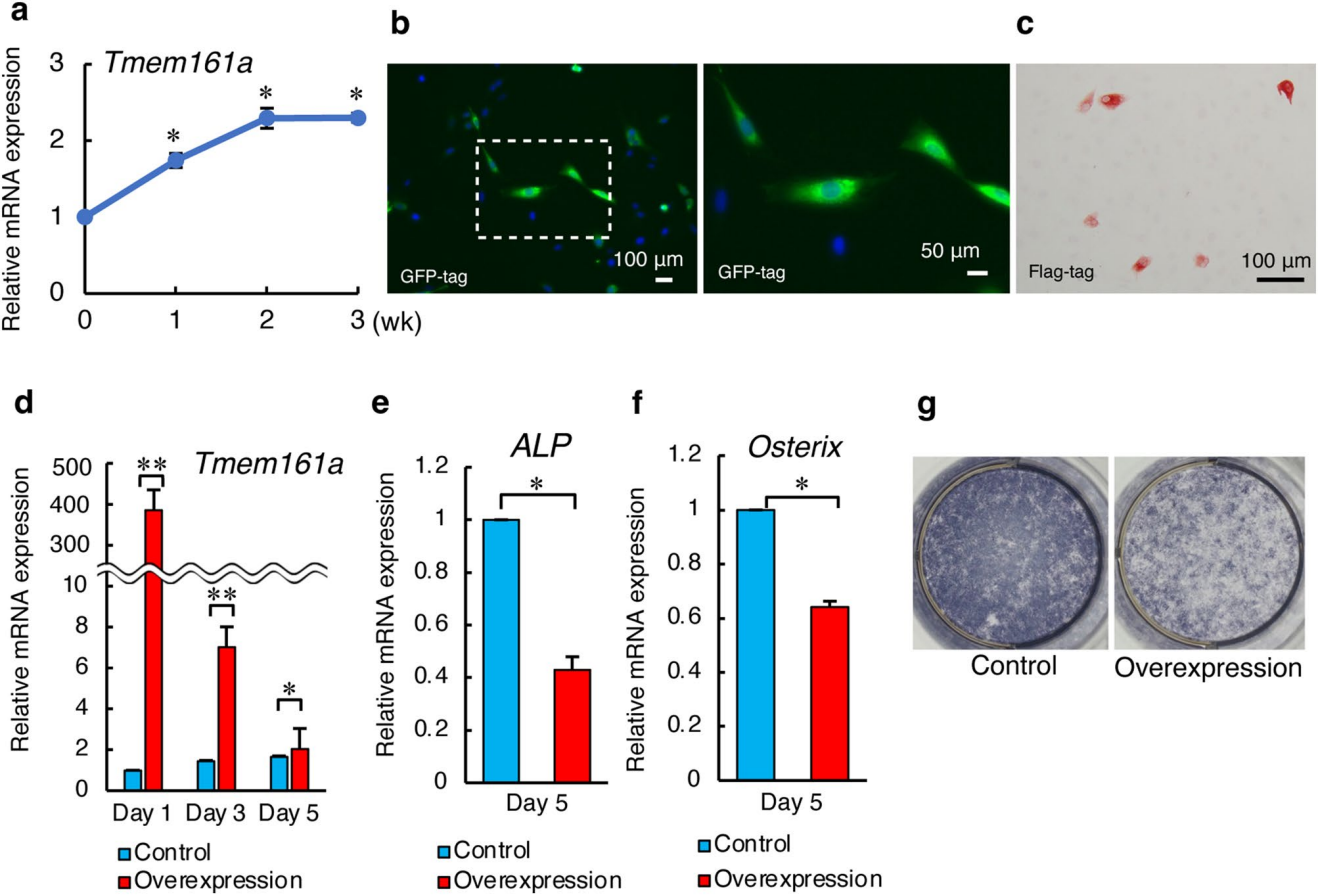
Osteoblast differentiation in Tmem161a-overexpressing MC3T3-e1 cells. (**a**) Expression of *Tmem161a* in MC3T3-e1 cells during osteoblast differentiation. (**b**) Fluorescence detection of GFP-tagged Tmem161a 1 day after transfection. Cell nuclei were stained with DAPI. Scale bar 100 μm (right), 50 μm (left). (**c**) Immunocytochemistry analysis of Tmem161a after transfection with an overexpression vector. (**d**) qPCR analysis of *Tmem161a* mRNA in MC3T3-e1 in control and Tmem161a-overexpression vector-transfected cells. qPCR analysis of *Alp* mRNA (**e**) and *Osx* mRNA (**f**). (**g**) ALP staining in MC3T3-e1 cells 5 days after transfection with control or Tmem161a-overexpression vectors. Data represent the mean ± SD from 3 independent experiments. Asterisks indicate statistically significant differences (*p < 0.05, **p < 0.01).

The functional importance of Tmem161a was studied in MC3T3-e1 cells, a murine osteoblastic cell line. In MC3T3-e1 cells cultured in osteogenic differentiation medium, *Tmem161a* mRNA expression was significantly increased on day 7 and 14 (Fig. 3a). Tmem161a was then transiently overexpressed in MC3T3-e1 cells. Successful transfection of the Tmem161a-overexpression vector was confirmed by GFP-staining (Fig. 3b). Tmem161a-overexpressing cells were confirmed by immunocytochemistry analysis based on detection of a flag-tag incorporated into the overexpression vector (Fig. 3c). Although *Tmem161a* mRNA expression was highest on day 1, overexpression was maintained until day 5 (Fig. 3d). In *Tmem161a*-overexpressing cells, expression of the alkaline phosphatase (*Alp*) gene, a marker of bone formation, was significantly decreased (Fig. 3e)*.* Expression of *Osx*, which encodes an essential transcription factor for osteoblast differentiation and bone mineralization, was also significantly decreased in Tmem161a-overexpressing cells (Fig. 3f). Significantly weaker ALP staining demonstrated reduced osteoblastic function in *Tmem161a*-overexpressing cells (Fig. 3g).

### Tmem161a does not affect on osteoclast differentiation

To confirm the effect of Tmem161a in osteoclast differentiation, co-cultures were performed using Tmem161a bone marrow cells and MC3T3-e1 cells. There was no significant difference in *Nfatc1* and tartrate-resistant acid phosphatase (*TRAP*) expression reflecting osteoclast function (Supplementary Fig. S5a,b). After cells were stained with TRAP, the concentration of staining was analyzed using Integrated Density of imageJ, there was no significant difference (Supplementary Fig. S5c–e).

### Depletion of *Tmem161a* promotes osteoblast differentiation

**Figure 4:**
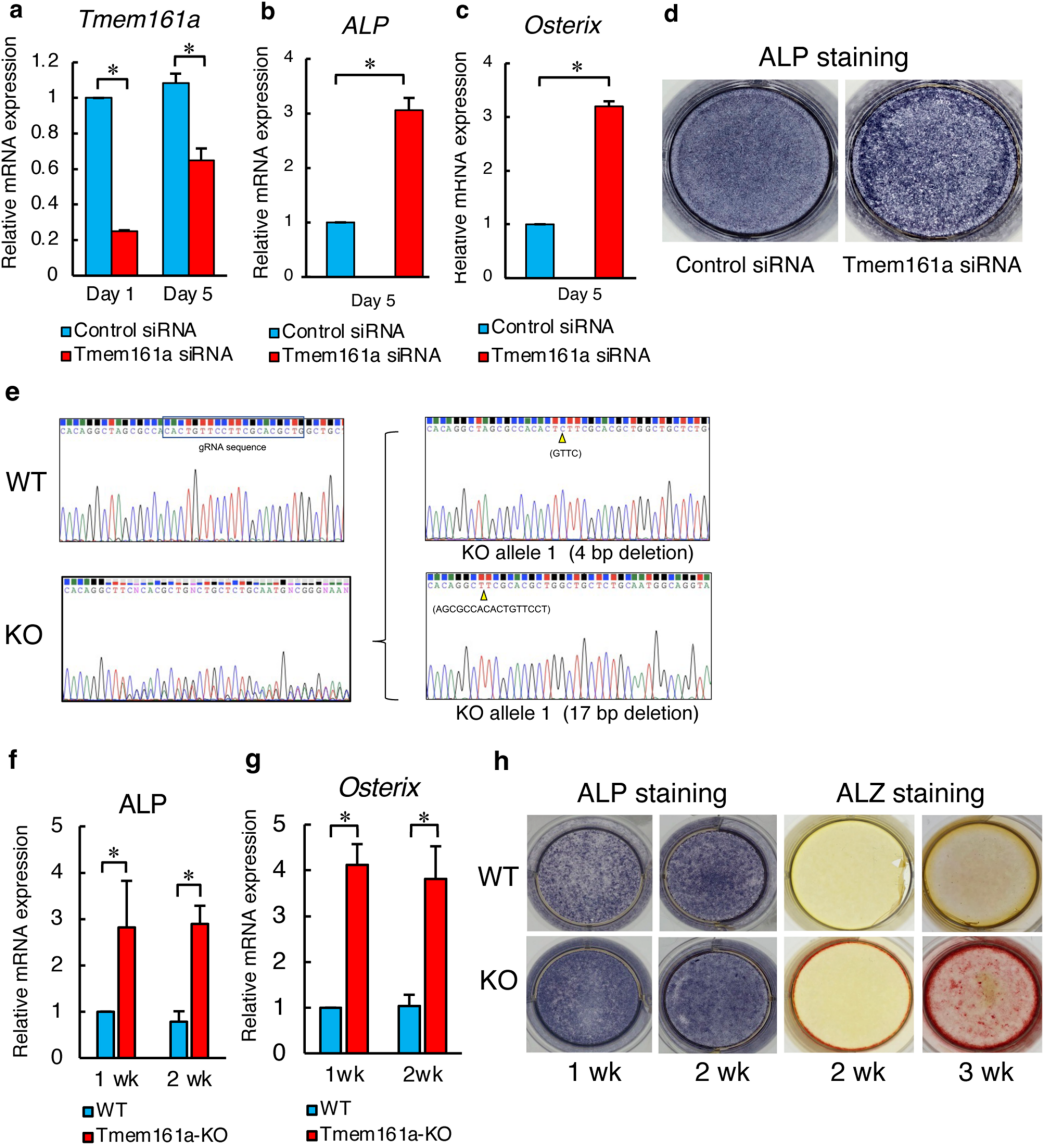
Osteoblast differentiation in Tmem161a-knockdown and -KO MC3T3-e1 cells. (**a**) qPCR analysis of *Tmem161a* mRNA in control and Tmem161a siRNA-transfected cells. qPCR analysis of *Alp* mRNA (**b**) and *Osx* mRNA (**c**)*.* (**d**) ALP staining in MC3T3-e1 cells 5 days after transfection. Osteoblast differentiation in Tmem161a-KO MC3T3-e1 cells generated using CRISPR/Cas9. (**e**) Sequencing of WT and KO cells. KO cells exhibited 4–base pair and 17–base pair deletions. qPCR analysis of *Alp* mRNA (**f**) and *Osx* mRNA (**g**) in WT and Tmem161a-KO cells during osteogenesis. (**h**) Staining of ALP and Alizarin Red S. Data represent the mean ± SD from 3 independent experiments. Asterisks indicate statistically significant differences (*p < 0.05).

To confirm the in vivo experimental findings, *Tmem161a*-knockdown experiments were performed using MC3T3-e1 cells. The efficiency of *Tmem161a* knockdown was 75% and 40% on day 1 and day 5, respectively (Fig. 4a). Moreover, expression of *Alp* and *Osx* mRNAs was significantly increased in Tmem161a-knockdown cells (Fig. 4b,c). Intense ALP staining in Tmem161a-knockdown cells indicated the promotion of osteoblast differentiation (Fig. 4d).

The Tmem161a-knockdown model is limited to the study of long-term effects, such as mineral deposition. Therefore, we generated Tmem161a-KO MC3T3-e1 cells using CRISPR/Cas9 (Supplementary Fig. S3a). Sequencing results revealed 4- and 17-base deletions, confirming successful generation of Tmem161a-KO cells (Fig. 4e, Supplementary Fig. S3b). The mRNA expression of osteoblast differentiation markers, such as *Alp* and *Osx*, was significantly increased in Tmem161a-KO cells compared to WT cells (Fig. 4f,g). Intense ALP and alizarin red S staining in Tmem161a-KO cells indicated increases in osteoblastic differentiation and calcium deposition, respectively (Fig. 4h).

### Tmem161a is involved in P38 MAPK signaling

**Figure 5:**
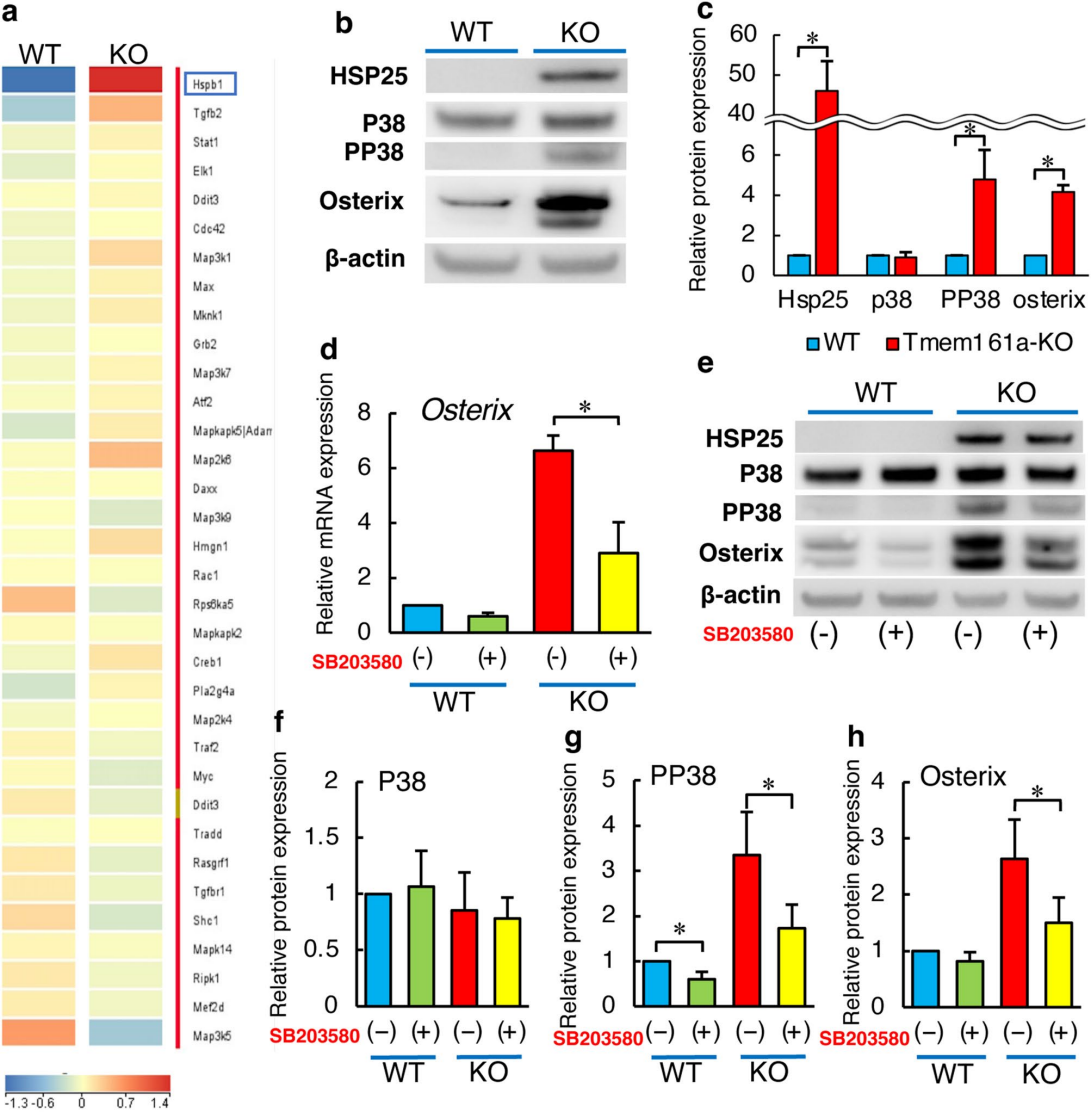
Activation of the P38 MAPK pathway in Tmem161a-KO cells. (**a**) Heatmap of WT and Tmem161a-KO cells. Microarray results were analyzed using Transcriptome Analysis Console software (version 4.0.2.15, ThermoFisher Scientific). (**b**) Western blotting of HSP25, P38, PP38, OSX, and β-actin in WT and Tmem161a-KO cells. (**c**) Densitometry analysis of HSP25, P38, PP38, and OSX expression. (**d**) *Osx* expression in WT and Tmem161a-KO cells with or without SB203580 treatment. (**e**) Western blotting of HSP25, P38, PP38, OSX, and β-actin in WT and Tmem161a-KO cells with or without SB203580 treatment. Densitometry analysis of P38 (**f**), PP38 (**g**), and OSX (**h**) in WT and Tmem161a-KO cells with or without SB203580 treatment. Data represent the mean ± SD from 3 independent experiments. Asterisks indicate statistically significant differences (*p < 0.05).

Microarray analysis of WT and Tmem161a-KO cells was used to screen for the expression of genes related to Tmem161a expression (Fig. 5a). Pathway analysis revealed upregulation of mitogen-activated protein kinase (MAPK) genes in Tmem161a-KO cells, especially heat shock protein b1 (*Hspb1*), *MAP3K1*, and *MAP2K6*. For more-detailed analysis, western blotting was performed to investigate the expression of HSP25, which is a product of the *Hspb1* gene (Fig. 5b). Previous studies indicated that *Hspb1* is closely associated with the P38 MAPK signaling cascade^14,15^. Therefore, we examined the expression of P38, phosphorylated P38 (PP38), and OSX. Densitometry analysis revealed significant increases in HSP25, PP38, and OSX expression in Tmem161a-KO cells compared with WT cells (Fig. 5c, Supplementary Fig. S4). To confirm the functional association between Tmem161a and P38 MAPK signaling, we treated cells with SB203580, an inhibitor of the P38 MAPK cascade^16,17^. In Tmem161a-KO cells treated with SB203580, *Osx* mRNA expression was significantly decreased (Fig. 5d). The effect of SB203580 was also examined by western blotting (Fig. 5e). Inhibition of the P38 MAPK cascade decreased the expression of PP38 and OSX in Tmem161a-KO cells, although it was higher than in WT cells. (Fig. 5f–h). The expression of HSP25 was not reduced by P38 inhibition.

### Increased ROS activity in Tmem161a-KO cells

**Figure 6:**
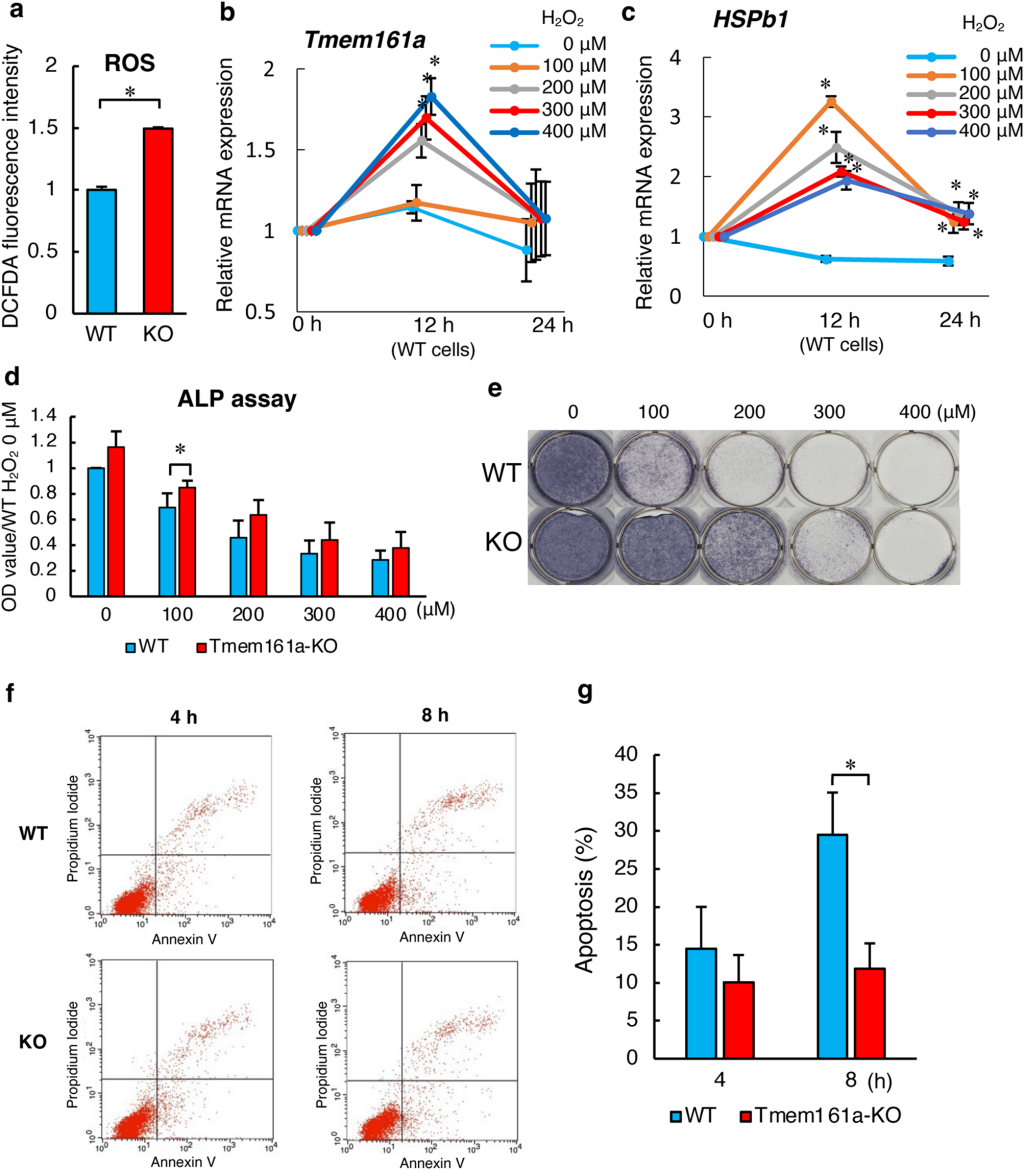
(**a**) ROS levels measured using DCFDA in WT and Tmem161a-KO cells. qPCR analysis of *Tmem161a* mRNA (**b**) and *Hspb1* mRNA (**c**) expression in response to oxidative stress induced by exposure to various concentrations of H_2_O_2_. ALP assay (**d**) and ALP staining (**e**) in WT and Tmem161a-KO cells after oxidative stress induction by exposure to various concentrations (0–400 μM) of H_2_O_2_ for 1 h. (**f**) Apoptotic cells detected based on annexin V using FACS analysis. (**g**) Number of apoptotic cells. Data represent the mean ± SD from 3 independent experiments. Asterisks indicate statistically significant differences (*p < 0.05).

P38 MAPKs are members of the MAPK family, and they are activated by a variety of environmental stresses, including oxidative stress^18,19^. Intracellular reactive oxygen species (ROS) stress was examined using an ROS assay kit, which revealed significantly increased cellular ROS levels in Tmem161a-KO cells (Fig. 6a). We then examined the effect of oxidative stress induced by exposure to various concentrations (0–400 μM) of hydrogen peroxide (H_2_O_2_) in WT MC3T3-e1 cells. Expression of *Tmem161a* mRNA increased in a concentration-dependent manner (Fig. 6b). In contrast, the highest expression of *Hspb1* mRNA was observed with 100 μM H_2_O_2_ (Fig. 6c). Moreover, ALP assay (Fig. 6d) and staining (Fig. 6e) demonstrated increased osteoblastic differentiation in Tmem161a-KO cells compared with WT cells. Flow cytometry revealed that Tmem161a-KO cells were resistant to apoptosis at 8 h after induction of oxidative stress (Fig. 6f,g).

## Discussion

This is the first study to investigate the role of Tmem161a in bone structure and function. The major findings of this study were that depletion of Tmem161a resulted in an increase in BMD, in turn leading to thickened cortical bones and enhanced bone strength. Moreover, Tmem161a is involved in osteoblast differentiation through the P38 MAPK pathway. These results indicate that Tmem161a plays an essential role in osteogenic differentiation.

Bone remodeling is a dynamic process that maintains the balance between bone formation by osteoblasts and bone resorption by osteoclasts^20^. A number of molecules are involved in the regulation of osteoblasts and osteoclasts, and their disruption leads to bone metabolic diseases, such as osteoporosis^21^. To identify novel genes involved in bone metabolism, we developed an efficient screening system using mutant mouse strains registered with the Exchangeable Gene Trap Clones database^9,10,22^. This screening process identified *Tmem161a* as a gene involved in bone metabolism. In our study, X-gal staining revealed ubiquitous expression of Tmem161a throughout the body. Detailed analyses indicated Tmem161a is expressed in articular cartilage, growth plates, and cortical bones. Immunohistochemical localization of Tmem161a in osteoblasts indicated it has functional importance in osteogenic differentiation.

Depletion of Tmem161a led to significantly stronger bone due to increased cortical bone thickness and BMD. A similar phenotype was observed in sclerostin-KO mice, a cystine-knot protein mainly expressed in bone^23^. Sclerostin-KO mice exhibited no difference in body weight (BW) or other phenotypes; but, bone mass and BMD were significantly increased in male and female mice compared with WT gender-matched littermates. However, Tmem161a-KO mice differed from sclerostin-KO mice in that it affects only cortical bone. In another study, the cortical bone mass and BMD were increased in myostatin-KO mice^24^. Myostatin, a member of the transforming growth factor family, is involved in the regulation of skeletal muscle growth. Depletion of myostatin induces an approximate doubling of skeletal muscle mass as well as 20% increase in cortical bone mineral content. Cortical bone is a dense external layer of bone critical for weight bearing due to its high resistance to bending and torsion^25^. Cortical bone is characterized by stiffness and high resistance to mechanical stress compared with trabecular bone^26^. In our study, depletion of Tmem161a also did not affect growth or BW of mice; however, the cortical bones became thicker and stronger. Collectively, these results indicate that Tmem161a is an important regulator of cortical bone mass and strength.

The direct evidence of the involvement of Tmem161a in osteogenic differentiation was confirmed by in vitro experiments. ALP is a glycoprotein secreted by osteoblasts that produces a high phosphate concentration on the osteoblast cell surface during bone mineralization^27,28^. *Osx* is an essential transcription factor for osteoblast differentiation and bone mineralization^29^. Therefore, both ALP and OSX could be used as reliable osteogenic markers of bone formation and bone calcification. In our study, Tmem161a overexpression decreased *Alp* and *Osx* expression in MC3T3-e1 cells. Conversely, increased *Alp* and *Osx* expression was observed in Tmem161a-knockdown or -KO cells. Alizarin Red S staining was increased in Tmem161a-KO cells, suggesting enhanced bone mineral deposition. Collectively, these results indicate that Tmem161a exerts a suppressive function in osteogenic differentiation during normal homeostasis. Although many studies have shown osteoclast function and osteoblast-osteoclast communication regulate bone remodeling, the results of co-cultures of mouse bone marrow cells and osteoblasts suggest that Tmem161a is more strongly associated with osteoblasts than with osteoclasts. However, the effect of Tmem161a on the coupling action of osteoclasts and osteoblasts still needs to be analyzed^30,31^.

Depletion of Tmem161a promoted the activation of P38 MAPK signaling, which is considered a stress-activated protein kinase pathway. An appropriate response to environmental and intracellular stresses is essential for homeostasis and survival^32^. In osteoblasts, activation of P38 induces increased expression of OSX and promotes osteogenesis^16,33,34^. The importance of P38 has also been confirmed in vivo, as deletion of P38a does not affect osteoclast function. Decreased expression of osteoblast-specific transcription factors and their targets results in impaired osteoblastogenesis, osteoblast maturation and activity^35^. The decreased expression of PP38 and OSX in Tmem161a-KO cells upon treatment with SB203580^16,17^, an inhibitor of P38, suggests that the loss of Tmem161a function increases OSX expression via activation of P38.

Excessive oxidative stress reportedly induces apoptosis of osteoblasts and suppresses ALP activity and matrix mineralization^36–38^. However, ROS can also have a positive stimulatory effect on bone regenerarion^39,40^. Pre-incubation of bone marrow stem cells in medium with low concentrations of H_2_O_2_ reduces apoptosis and promotes cell survival^41^. ROS assays and incubation with H_2_O_2_ revealed anti-oxidative stress effects in Tmem161a-KO cells. Furthermore, the induction of HSP25 suggested that these cells are resistant to oxidative stress. In the absence of Tmem161a, activation of P38 MAPK signaling resulted in upregulation of *Alp* and *Osx*. In Tmem161a-KO cells, the activation of P38 MAPK signaling and increased expression of OSX were abolished by treatment with the selective inhibitor SB203580, suggesting that Tmem161a plays an important role in mediating P38 MAPK signaling in osteoblasts. The loss of Tmem161a enhances osteoblast function through phosphorylation of P38 and enhanced transcription of *Osx*.

In summary, we demonstrated that Tmem161a plays an important role in osteogenic differentiation using in vivo and in vitro models. Depletion of Tmem161a induced stronger bone in mouse femurs due to thicker cortical bone and increased BMD. Tmem161a is important for tolerance against oxidative stress through P38 MAPK signaling.

## Materials and methods

### Animals and tissue preparation

All experiments using mice were performed with the approval of the Animal Care and Use Committee and the Genetic Modification Safety Committee of Kumamoto University and the University of Miyazaki (2015-513-5, 612), in accordance with the Institutional Guidelines for Animal Experiments and Safety Management Rules for Genetic Modification. The ARRIVE Essential 10 guideline was used to formulate the study design, sample preparation, results observation, and data analysis. Mice were euthanized with intra-peritoneal overdose injection of thiamylal sodium (Nichi-Iko Pharmaceutical, Tokyo, Japan). *Tmem161a*^*GT/GT*^ mice were generated using the exchangeable Gene Trap system^42^. Femurs were obtained from 8-week-old WT and *Tmem161a*^*GT/GT*^ mice and then snap frozen until qPCR analysis. For morphological and immunohistochemical analyses, femurs were fixed overnight in 4% paraformaldehyde (PFA) in phosphate-buffered saline (PBS) at room temperature (RT) and subsequently embedded in paraffin using standard methods. Five to ten mice were used in each experimental group.

### Bone morphometric analysis

Femurs were obtained from 8-week-old WT and *Tmem161a*^*GT/GT*^ male mice and analyzed by μCT using a μCT system (ScanXmate-L090H; Comscantecno, Kanagawa, Japan), as described previously^10^. Bone morphology was analyzed using a three-dimensional image analysis system (TRI/3D-BON; RATOC System Engineering, Tokyo, Japan) (n = 10)^10^. For cortical bone analyses, the mid-point of the femur was scanned. For trabecular bone analyses, the measurement area was set as 0.2 mm from the growth plate to 2 mm into the proximal area. Bone morphometric analyses included cortical C.BMD, cortical volume (Ct.V), Cortical bone thickness Ct.Th, trabecular bone mineral density (T.BMD), and bone volume (BV), Trabecular bone thickness (Tb.Th), Trabecular number (Tb.N).

### Biomechanical strength analysis

Bone strength of the femoral shaft was determined using a three-point bending test (EZ-test S; Shimadzu Co., Kyoto, Japan), as described previously^9^. The span between the two support points was 6 mm. Maximum load (M.work, N), maximum stress (M.stress, N/mm^2^), and maximum displacement (M.dsp, mm) were measured until the sample broke at a test speed of 1 mm/min.

### X-gal staining

X-gal staining was performed using the method described by Allen^43^*.* The samples were fixed in 1% formaldehyde, 0.2% glutaraldehyde, and 0.02% NP-40 in PBS at RT. After 30 min of agitation, the samples were transferred into staining solution (5 mM potassium ferricyanide, 5 mM potassium ferrocyanide, 2 mM MgCl_2_, 0.5% X-gal in PBS) and incubated overnight at RT.

### Immunohistochemistry

To examine Tmem161a expression in bone, paraffin-embedded femur tissues of 8-day-old mice without demineralization were used. The sections were deparaffinized with toluene, rehydrated using a graded ethanol series, and then microwaved at 95 °C for 15 min in 10 mM citrate buffer (pH 6.0). After inhibition of endogenous peroxidase activity by incubation in 0.3% H_2_O_2_ in methanol for 15 min, the sections were reacted with 500 µg/ml normal goat IgG and 1% bovine serum albumin/PBS for 1 h to block non-specific binding of antibodies. The sections were then reacted with anti-Tmem161a antibody (ThermoFisher Scientific, Waltham, MA, USA) overnight. After washing with 0.075% Brij L23 in PBS, the sections were reacted with HRP-goat anti-rabbit IgG for 1 h. After washing in 0.075% Brij L23 in PBS, the HRP-sites were visualized by incubation in DAB, Ni, Co, and H_2_O_2_ solution for 5 min^44^.

### Cell culture

MC3T3-e1 murine osteoblasts were purchased from RIKEN (RIKEN BioResource Center, Tsukuba, Japan). The cells were cultured in α-minimum essential medium (α-MEM) supplemented with 10% fetal bovine serum and penicillin–streptomycin-amphotericin B in the presence of 5% CO_2_ at 37 °C. To induce osteoblastic differentiation, the cells were incubated in osteogenic induction medium, which consisted of α-MEM, 10 mM β-glycerophosphate, and 50 μg/ml ascorbic acid. The medium was changed once every 2–3 days.

### Tmem161a-knockdown experiments

MC3T3-e1 murine osteoblasts were divided into two groups. The cells were transfected with control siRNA (#4390843, ThermoFisher Scientific) or Tmem161a siRNA (#4390771, ThermoFisher Scientific) using lipofectamine (RNA iMAX, Invitrogen, CA, USA). MC3T3-e1 cells were seeded at 50,000 cells/well in a 24-well plate and incubated in osteogenic differentiation medium. The medium was changed on day 3, and the cells were analyzed on day 5.

### Transient overexpression of Tmem161a

MC3T3-e1 cells were electroporated using a Tmem161a-GFP-tagged plasmid vector (#MG207689, OriGene, Rockville, MD, USA) and Tmem161a-Flag-tagged plasmid vector (#MR207689, OriGene) or empty vector using NEPA21 (Nepa Gene, Chiba, Japan). The electroporation conditions were pulse voltage = 175 V, pulse interval = 50 ms, pulse width = 50 ms, and pulse number = 2. The differentiation medium was changed on day 3, and the cells were evaluated on day 5.

### Generation of Tmem161a-KO cells

Single guide (sg)RNAs were constructed using a GeneArt precision gRNA synthesis kit (ThermoFisher Scientific) with forward primer 5ʹ-TAATACGACTCACTATAGCAGCGTGCGAAGGAAC-3ʹ and reverse primer: 5ʹ-TTCTAGCTCTAAAACCACTGTTCCTTCGCACGCT-3ʹ. The PAM sequence was located within *Tmem161a* exon2 and designed as CAGCGTGCGAAGGAACAGTG.

MC3T3-e1 cells were transfected with the generated sgRNA, Lipofectamine CRISPR max reagent (ThermoFisher Scientific), and True cut cas9 protein V2 (ThermoFisher Scientific). The KO cell line was then established using the limited dilution method in a 96-well plate. Mutant sequences were confirmed by direct sequencing. The KO cells were cloned using a Zero Blunt TOPO PCR cloning kit (ThermoFisher Scientific) and XL-1 blue (Agilent Technologies, Santa Clara, CA, USA), followed by sequencing analysis.

### ALP and alizarin red S staining

MC3T3-e1 cells were fixed in 4% PFA for 10 min. For ALP staining, after washing three times with PBS, the cells were incubated with reagents of a TRAP/ALP staining kit (Fujifilm Wako Chemicals, Osaka, Japan) for 30 min at 37 °C. For ARS staining, after washing three times with double-distilled water (DDW), the cells were incubated with 1% ARS solution (Muto, Tokyo, Japan) for 5 min at RT.

### ALP assay

MC3T3-e1 cells were seeded in a 96-well plate at a density of 5000 cells/well and incubated for 24 h. The culture medium was then replaced with osteogenic induction medium with or without H_2_O_2_. The medium was changed every 2 days. After 7 days of osteogenic differentiation, ALP activity was measured using a TRAP and ALP assay kit (Takara, Shiga, Japan). The absorbance of each well was measured at 405 nm using a Spectramax iD5 spectrophotometer (Molecular Devices, Tokyo, Japan).

### Western blotting

Protein was extracted from cultured MC3T3-e1 cells using RIPA buffer. Cell lysates containing 20 µg of protein were mixed with sample buffer solution (Fujifilm) and separated on NuPAGE 4–12%® Bis–Tris Gels (ThermoFisher Scientific). Proteins were electrophoretically transferred onto iBlot 2 membranes (ThermoFisher Scientific) for dry protein transfer and then washed with DDW. The membranes were blocked with 4% Block Ace (KAC Co., Ltd., Japan) at RT for 1 h. The membranes were then incubated overnight at 4 °C with rabbit polyclonal anti-Hsp25 antibody (ab202846, Abcam, Tokyo, Japan), rabbit polyclonal anti-p38 MAPK antibody (#9212, Cell Signaling, MA, USA) 1:2000, rabbit polyclonal anti–phospho-p38 MAPK antibody (#9211, Cell Signaling) 1:800, rabbit monoclonal anti-Sp7/OSX antibody (ab209484, Abcam) 1:1,000, or mouse monoclonal anti–β-actin antibody (A3854, Sigma-Aldrich, Tokyo, Japan) 1:200,000. Membranes were then reacted with HRP-goat anti-rabbit IgG antibody (DK-2600, Dako, Glostrup, Denmark) 1:5000 for 1 h. Protein bands were visualized by chemiluminescence using an ImageQuant LAS4000 imaging system (GE Healthcare, Fairfield, CT, USA). Densitometric analysis was performed using ImageQuant LAS 4000 software. β-Actin was used as an internal standard in each lane for normalization of target protein expression.

### RT-PCR

RNA was extracted from femur bone using Isogen (Nippongene, Tokyo, Japan) and then converted to cDNA using Moloney murine leukemia virus reverse transcriptase (Invitrogen). qPCR was performed using Ex taq (Takara) polymerase and primer sets as listed below. RNA was also extracted from MC3T3-e1 cells using a Reliaprep RNA Miniprep system (Promega, Madison, WI, USA) according to the manufacturer’s protocol. qPCR was performed on a StepOne™ Real-time PCR system (Applied Biosystems, Waltham, MA, USA) using Fast SYBR Green master mix (ThermoFisher Scientific). The relative expression of target genes was calculated using the 2^−ΔΔCT^ method. The expression profiles of *Tmem161a*, *Alp*, *Osx*, *Hspb1*, and *β-actin* were evaluated. The primer pairs used in the experiments are listed below. Tmem161a Intron1: F-5ʹ-CACTGACTTCTGAGAGATACC-3ʹ (primer a of Fig. 1a), Tmem161a Exon2: R-5ʹ-CTGCCATTGCAGAGCAG-3ʹ (primer b), pU21 SA14AS: R-5ʹ- GAGTAGGAGTCGTACCTACTC-3ʹ (primer c), Tmem161a Exon1: F-5ʹ-CCCAGGGAGCGGACATG-3ʹ (primer d), pU21 Z3 R-5ʹ-TCTACTGCTACGTGACTGTGG-3ʹ (primer e), Tmem161a Exon11 F-5ʹ-TCTACTGCTACGTGACTGTGG-3ʹ (primer f), Tmem161a Exon12: R-5ʹ-CACCAGATGATGTAAGCCAG-3ʹ (primer g). For qPCR, Tmem161a F-5ʹ-CCAGTCCAGGAGACCAACATC-3ʹ, R-5ʹ-GAGACATACGGAGCGCTCAC-3ʹ, *Alp* F-5ʹ-CCAACTCTTTTGTGCCAGAGA-3ʹ, R-5ʹ-GGCTACATTGGTGTTGAGCTTTT-3ʹ, *Osx* 5ʹ-ATGGCGTCCTCTCTGCTTG-3ʹ, R-5ʹ-TGAAAGGTCAGCGTATGGCTT-3ʹ, *Hspb1* 5ʹ-GCTCACAGTGAAGACCAAGG-3ʹ, R-5ʹ-GAAGCACCGAGAGATGTAGC-3ʹ, *β-actin* 5ʹ-GAGCTATGAGCTGCCTGACG-3ʹ, R-5ʹ-AGTTTCATGGATGCCACAGG-3ʹ.

### ROS assay

Cellular ROS were detected using a DCFDA/H2DCFDA-Cellular ROS assay kit (Abcam, Cambridge, UK). The cells were seeded into 96-well plates at a density of 25 × 10^3^ cells/well and cultured for 24 h. After washing, the cells were incubated in 10 μM DCFDA for 45 min in the dark. Absorbance was measured at Ex/Em = 485/535 nm using a Spectramax iD5 spectrophotometer (Molecular Devices).

### Microarray

RNA was isolated from WT and Tmem161a-KO cells generated using the CRISPR/Cas9 system (n = 3 each), and then cRNAs were prepared as described previously^45^. A total of 15 µg of fragmented cRNA from each sample was hybridized to a pre-equilibrated Clariom S mouse microarray chip, which was then washed, stained, and scanned using an HP ChipScanner (Affymetrix Inc., Santa Clara, USA). Data normalization was performed using GeneSpring (Agilent Technologies). All entities (22,206 genes) were filtered based on significant changes in gene expression between WT and Tmem161a-KO cells. Data were analyzed in Transcriptome Analysis Console software (version 4.0.2.15, ThermoFisher Scientific).

### Flow cytometry

Cell apoptosis was assessed using an Annexin V-FITC apoptosis detection kit (Nacalai Tesque, Kyoto, Japan) with FACS. Briefly, cells were seeded into 6-cm dishes at a density of 21 × 10^4^ cells/well and incubated for 24 h. The cells were then treated with 400 μM H_2_O_2_. After 4 and 8 h of H_2_O_2_ treatment, adherent and floating cells in each well were harvested and washed with PBS. A 100-μl aliquot of cells was incubated with 5 μl of Annexin V-FITC conjugate and 5 μl of propidium iodide for 30 min at RT in the dark. Apoptotic cells were identified by flow cytometry using a BD FACS Calibur.

### Statistical analysis

Data were analyzed by Student's *t*-test using Statistical Package for Social Sciences software (ver. 20; IBM Corp., Armonk, NY, USA). The data shown in graphs are presented as the mean ± standard deviation, and p values of < 0.05 were considered to indicate statistical significance.

### Supplementary Information

Supplementary Information.

## Data Availability

Original micrographs and any other information are available upon request from the corresponding author. The datasets generated and analysed during the current study are available in the GEO repository, [https://www.ncbi.nlm.nih.gov/geo/query/acc.cgi?acc=GSE227961]”.
