# Supplementary material for: Tmem161a regulates bone formation and bone strength through the P38 MAPK pathway
Source: Sci Rep. 2023 Sep 5;13:14639. doi: 10.1038/s41598-023-41837-4 (PMC10480474; doi:10.1038/s41598-023-41837-4)
Supplement: Supplementary file 1 — Supplementary Information. [file 41598_2023_41837_MOESM1_ESM.pdf]

Tmem161a regulates bone formation and bone strength through the P38 MAPK pathway

Takuya Nagai[1], Tomohisa Sekimoto[1], Syuji Kurogi[1], Tomomi Ohta[1], Shihoko Miyazaki[1], Yoichiro Yamaguchi[1], Takuya Tajima[1], Etsuo Chosa[1], Mai Imasaka[2], Kumiko Yoshinobu[3], Kimi Araki[3], Masatake Araki[3], Narantsog Choijookhuu[4], Katsuaki Sato[5] Yoshitaka Hishikawa[4] and Taro Funamoto[1*]

[1] Division of Orthopaedic Surgery, Department of Medicine of Sensory and Motor Organs, Faculty of Medicine, University of Miyazaki, Japan

[2] Department of Genetics, Hyogo Medical University, Japan

[3] Institute of Resource Development and Analysis, Kumamoto University, Japan

[4] Department of Anatomy, Histochemistry and Cell Biology, Faculty of Medicine, University of Miyazaki, Japan

[5] Division of Immunology Department of Infectious Disease, Faculty of Medicine, University of Miyazaki, Japan

Running title: Tmem161a regulates bone formation and bone strength.

*Corresponding author: Taro Funamoto MD., Ph.D.

Division of Orthopaedic Surgery, Department of Medicine of Sensory and Motor Organs, Faculty of Medicine, University of Miyazaki, 5200 Kihara, Kiyotake, Miyazaki 889-1692, Japan. Tel: +81-985-85-0986; Fax: +81-985-84-2931, E-mail: taro_funamoto@med.miyazaki-u.ac.jp

Supplementary figure S1

a

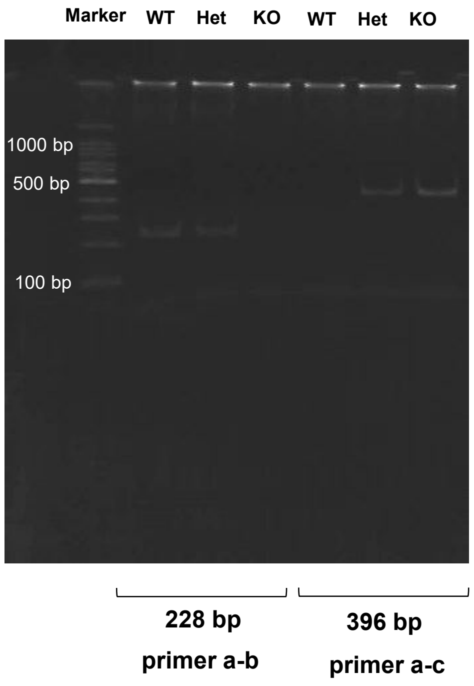

b

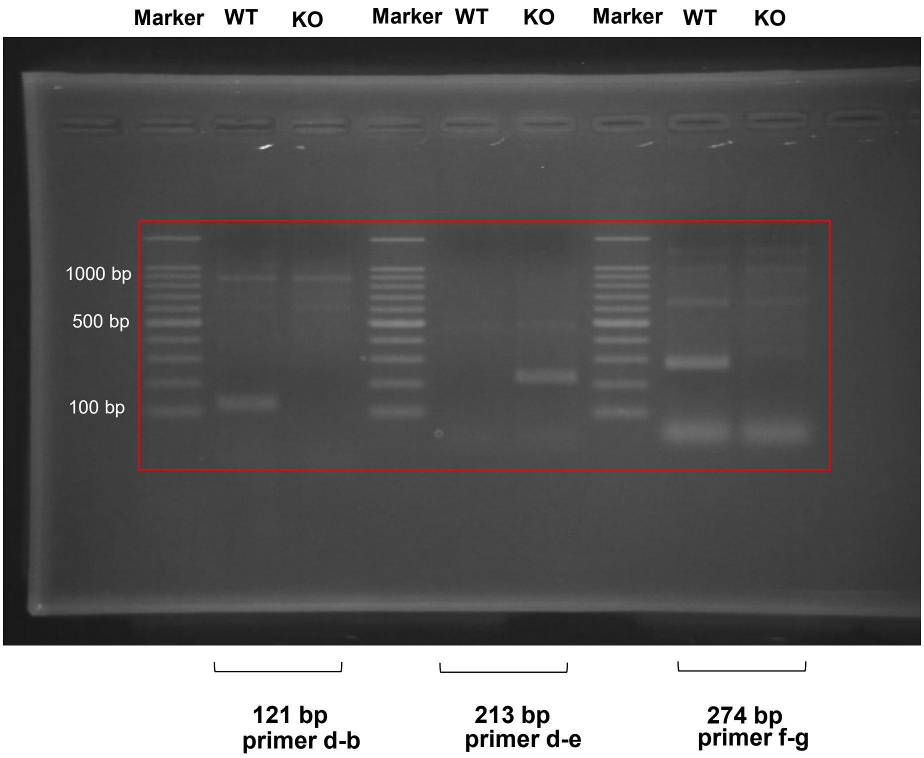

Supplementary figure S2

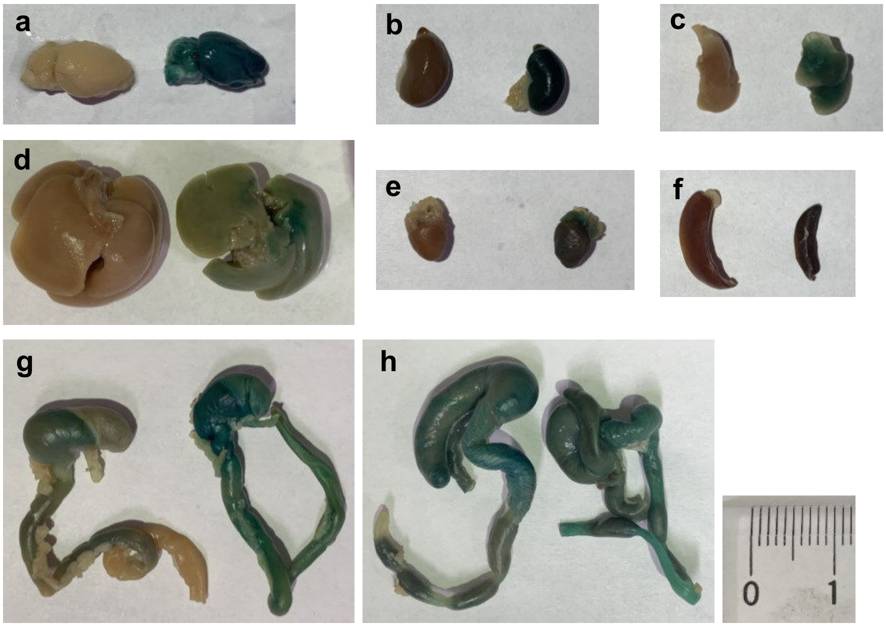

Left : WT Right : *Tmem161a$^{GT/GT}$*

Supplementary figure S3

a Tmem161a

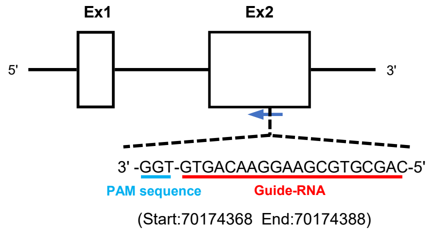

b

4 bp deletion

ATGGCGGTCCTTGGAGTGCAGCTGGTGGTGACTCTGTTCACCGCCACCCTCATGCACAGG
CTAGCGCCACACT(GTTC)CTTCGCACGCTGGCTGCTCTGCAATGGCAGTCTGTTCCGGTAC
ATACACCCGTCAGAGGAGGAACTTCGGGCGCTGTCAGGGAAAC**TGA**GGCCCCGGGTCAG
GAAGGAGCGGTGGGCAAATGGTCTTCATGACGAGAAGCCATTGTCAGT

17 bp deletion

ATGGCGGTCCTTGGAGTGCAGCTGGTGGTGACTCTGTTCACCGCCACCCTCATGCACAGG
CT(AGCGCCACACTGTTCCT)TCGCACGCTGGCTGCTCTGCAATGGCAGTCTGTTCCGGTAC
ATACACCCGTCAGAGGAGGAACTTCGGGCGCTGTCAGGGAAACTGAGGCCCCGGGTCAG
GAAGGAGCGGTGGGCAAATGGTCTTCA**TGA**CGAGAAGCCATTGTCAGTGCCTCGAGATGC
CCATTTCCAGCTGCAGACCTGTCCCCTCACTGCTGTGGATGCCCTA

Supplementary figure S4

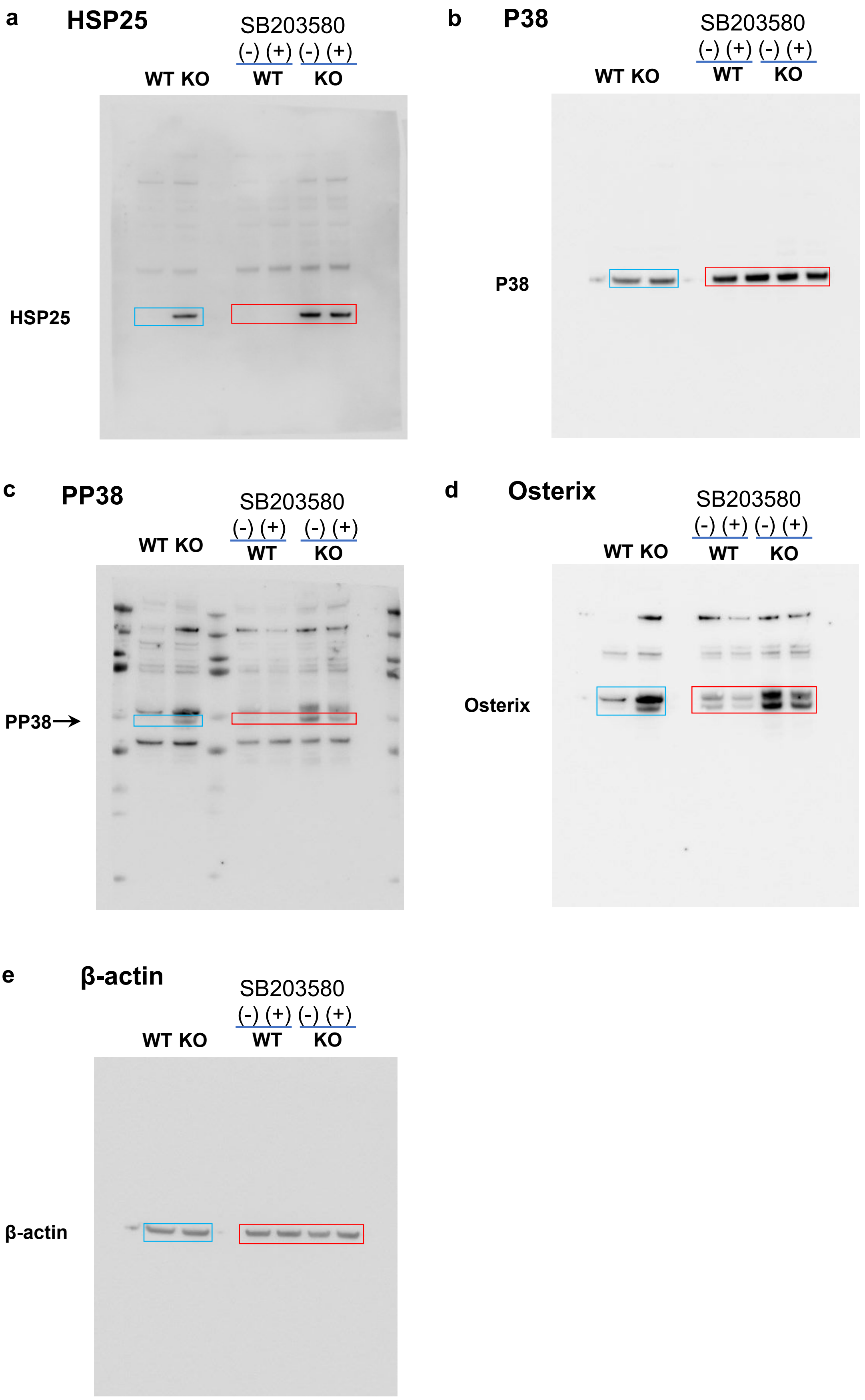

Supplementary figure S5

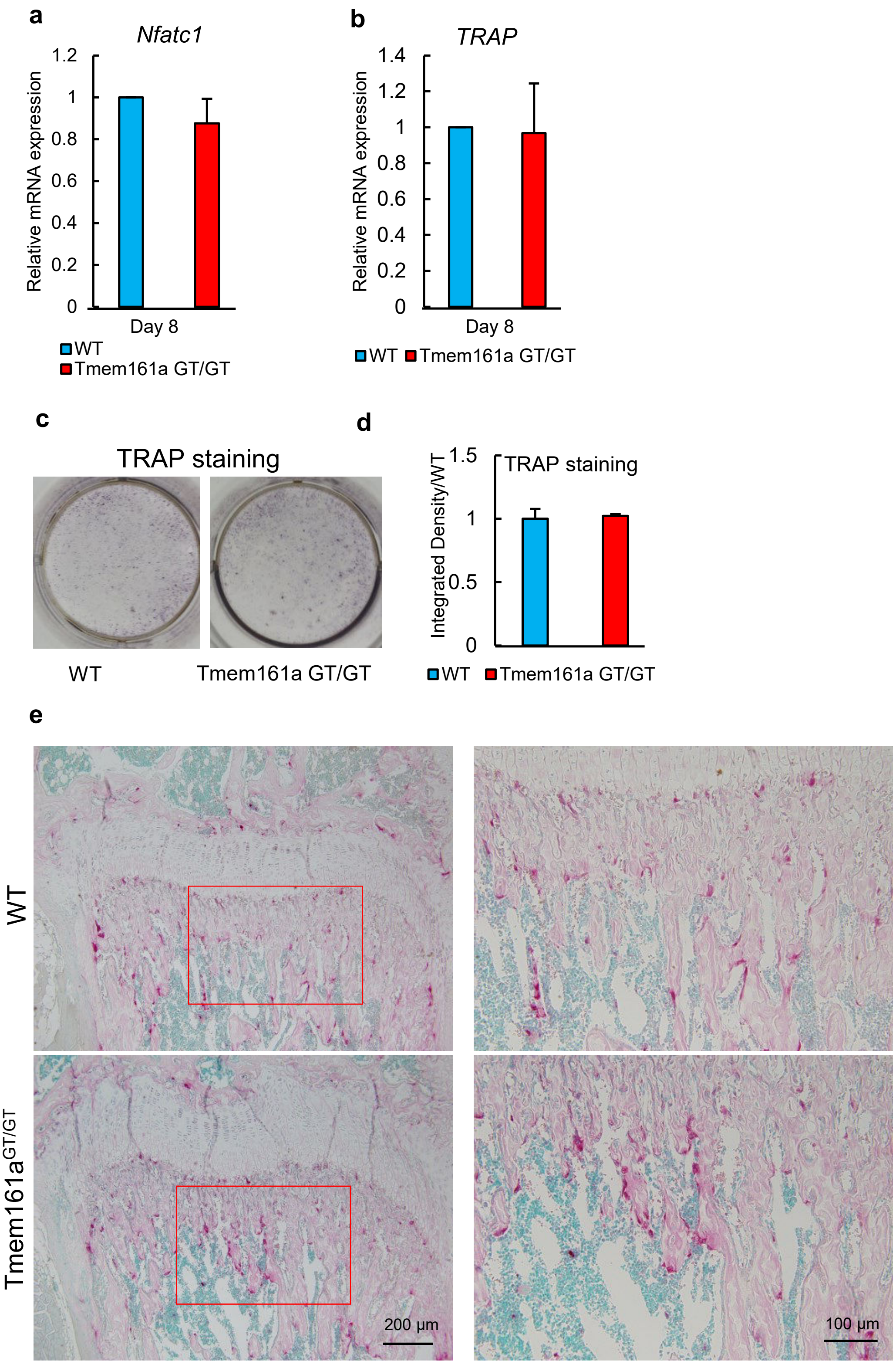

Supplementary Figure S1.

(**a**) Genomic-PCR analysis of *Tmem161a* in WT and *Tmem161a*$^{GT/GT}$ mouse bone tissues. Left band is between intron 1 and Exon2 (primer a and b). Right band is between intron1 and the trap vector (primer a and c). (**b**) Original image of RT-PCR that revealed null of Tmem161a in *Tmem161a*$^{GT/GT}$ mice. The red box shows Figure 1b.

Supplementary Figure S2.

X-gal staining of WT (left) and *Tmem161a*$^{GT/GT}$ (right) mouse tissues. Brain (**a**), kidney (**b**), lung (**c**), liver (**d**), heart (**e**), spleen (**f**), small intestine (**g**), and large intestine (**h**).

Supplementary Figure S3.

(**a**) Single guide RNA and PAM sequence of Tmem161a Exon2 for editing Tmem161a knockout cells. (**b**) Sequencing of Tmem161a-KO MC3T3-e1 cells generated using CRISPR/Cas9. sgRNA and Cas9 protein were inserted into MC3T3-e1 cells. Four– and 17–base pair deleted sequences and stop codons are highlighted in red and yellow, respectively.

Supplementary Figure S4.

Full membranes of western blotting analyses of HSP25 (**a**), P38 (**b**), PP38 (**c**), OSX (**d**), and β-actin (**e**) in WT and Tmem161a-KO cells with or without SB203580 treatment. The blue box shows Figure 5b and the red box shows Figure 5e. The membrane was stripped with Western Blot Stripping Buffer (Takara).

Supplementary Figure S5.

Osteoclast culture by co-culture of mouse bone marrow cells and MC3T3-e1.

qPCR analysis of *Nfatc1* mRNA (**a**) and *TRAP* mRNA (**b**). TRAP staining (**c**). Integrated Density/WT of TRAP staining (**d**) TRAP staining in 8-week-old WT and *Tmem161a*$^{GT/GT}$ mouse femurs (**e**). Data represent the mean ± SD from 3 independent experiments.

Supplementary materials and methods

Osteoclast culture by co-culture of mouse bone marrow cells and MC3T3-e1.

A mixture of 1.2 million bone marrow cells collected from mice bone marrow of femurs of 8-week-old WT and *Tmem161a*$^{GT/GT}$ male mice and 120,000 MC3T3-e1 were cultured with 10 nM 1α,25-DihydroxyvitaminD_3 and 1 μM Prostaglandin E2. The medium was changed on day 3, and the cells were analyzed on day 8. Cells were washed with PBS, fixed with 4% PFA at 37°C for 10 min, and incubated in the presence of TRAP solution (387A, Sigma-Aldrich, Tokyo, Japan) for 1 hr. The primer pairs used in the experiments are listed below. *Nfatc1* 5'-AGCCCAAGTCTCACCACAG-3', R-5'-AGCGTGAGAGGTTCATTCTC-3', *TRAP* 5'-TTCACTGGAGTGCACGATG-3', R-5'-ATGCAATCTGTGCAGAGACG-3'
